# Repetitive transcranial magnetic stimulation over primary motor vs non-motor cortical targets; effects on experimental hyperalgesia in healthy subjects

**DOI:** 10.1186/s12883-014-0166-3

**Published:** 2014-09-04

**Authors:** Paul Sacco, Michael Prior, Helen Poole, Turo Nurmikko

**Affiliations:** Pain Research Institute, Institute of Ageing and Chronic Disease, University of Liverpool, Liverpool, L9 7AL UK; The Walton Centre NHS Foundation Trust, Liverpool, UK; Faculty of Science, Liverpool John Moores University, Liverpool, UK

**Keywords:** Experimental pain, Capsaicin, Repetitive TMS

## Abstract

**Background:**

High frequency repetitive transcranial magnetic stimulation (rTMS) targetted to different cortical regions (primary motor/sensory, prefrontal) are known to alter somatosensory responses. The mechanism(s) for these effects are unclear. We compared the analgesic effects of rTMS at different cortical sites on hyperalgesia induced using topical capsaicin cream.

**Methods:**

Fourteen healthy subjects had capsaicin cream applied to a 16 cm^2^ area of the medial aspect of the right wrist (60 min) on 4 separate occasions over 6 weeks. rTMS (10Hz for 10s/min = 2000 stimuli @ 90% resting motor threshold of first dorsal interosseus muscle) was applied to the optimum site for right hand (M1), left dorsolateral prefrontal (DLFPC) and occipital midline (OCC) in a pseudo-randomised order. Thermal and mechanical perception and pain thresholds were determined using standardised quantitative sensory testing (QST) methods at the capsaicin site. Subjective responses to thermal stimuli (pain score on a numerical rating scale) from −2.5°C to +2.5°C of the individualised heat pain threshold (HPT) resulted in a hyperalgesia curve. Sensory testing took place prior to capsaicin application (PRE-CAP), after 30 min of capsaicin (POST-CAP) and following rTMS (30 min = POST-TMS).

**Results:**

Capsaicin application resulted in substantial changes in thermal (but not mechanical) sensitivity to both heat and cold (eg. HPT PRE-CAP = 43.6°C to POST-CAP = 36.7°C (p < 0.001)) with no differences between groups pre-rTMS. POST-TMS HPT showed no changes for any of the treatment groups, however the pain scores for the hyperalgesia curve were significantly lower for M1 vs OCC (−24.7%, p < 0.001) and for M1 vs DLFPC (−18.3%, p < 0.02).

**Conclusion:**

rTMS over the primary motor cortex results in a significant analgesic effect compared to other cortical areas.

**Electronic supplementary material:**

The online version of this article (doi:10.1186/s12883-014-0166-3) contains supplementary material, which is available to authorized users.

## Background

Since the first report of the beneficial effects of epidural motor cortex (M1) stimulation using implanted electrodes in patients with chronic post-stroke pain [1] many studies have confirmed the effectiveness of this approach in a variety of pain conditions [2,3]. The limitations and risk of complications of this approach have led to the use of non-invasive brain stimulation to treat patients with chronic pain, namely repetitive transcranial magnetic stimulation (rTMS) [4,5] and transcranial direct current stimulation (tDCS) [6]. The safety and ease of use of these techniques has allowed investigation of non-motor cortical areas such as pre-frontal cortex [7,8], or somatosensory areas [9,10] with mixed success. However, the optimal cortical targets for treating specific pain conditions as well as the potential mechanisms by which pain relief occur are unclear.

Various types of experimentally induced pain have been used to study the effects of non-invasive brain stimulation on somatosensory responses in healthy subjects. Summers et al. [11] found that rTMS over M1 reduced the temperature at which subjects reported a thermode applied to the hand was perceived as painful, but did not affect heat pain thresholds (suggesting an effect on nociceptive A-delta but not C-fibres). In contrast, Graff-Guerrero et al. [12] found no change in response to an arm cold pressor test following rTMS over M1, but a significant increase in pain tolerance when dorsolateral prefrontal cortex (DLPFC) was targetted. Further evidence supporting the analgesic effects of rTMS come from Nahmias et al. [13] who found that both M1 and rTMS stimulation significantly increased thermal pain thresholds in both hand and foot (although these were more pronounced in the cold domain). Another model of experimental pain in which rTMS has been used involves the use of the irritant capsaicin, either injected intradermally, or topically using a combination of heat and capsaicin to induce a transient heat hyperalgesia [14,15]. Tamura et al. [16] found that rTMS over M1 resulted in enhanced recovery from pain induced by capsaicin injection compared with sham rTMS or control. Brighina et al. [17] compared rTMS of the left and right DLFPC on pain responses to topical capsaicin application to both hands and found significant bilateral pain reductions after stimulating the left hemisphere only. It is not clear whether the analgesic effect of stimulation of motor and prefrontal cortices operate through similar pathways/mechanisms.

Therefore this study aimed to directly compare the efficacy of M1 vs left DFLPC stimulation in a topical capsaicin model of heat hyperalgesia, relative to a control rTMS condition (stimulation of the occipital fissure).

## Methods

### Subjects

Fourteen healthy volunteers (eight female/six male, mean age 23.6 years (18–51 year range)) gave informed written consent to take part. Participants had no clinical symptoms or signs of peripheral or central nervous system disorders and took no psychoactive medication. The study was approved by Liverpool John Moores University Faculty of Science ethics committee and performed in accordance with the declaration of Helsinki.

### Thermal detection and pain thresholds

Both thermal detection and pain thresholds were measured using Quantitative Sensory Testing (QST) via a MEDOC TSA −2001 Thermal Sensory Analyzer (MEDOC, Israel). The protocol for tests were based on the standardized QST procedures developed by the German Network on Neuropathic Pain [18]. A contact thermode which measured 25 mm × 25 mm was used to measure thermal responses at three sites: capsaicin site (CAPSITE) on the medial aspect of the right wrist; the near capsaicin site (nrCAPSITE) approximately 20 mm adjacent to the capsaicin site; and a contralateral capsaicin site (clCAPSITE) on the medial aspect of the left wrist. The baseline temperature of the contact thermode was 32°C (thermode range 0°C – 50°C). Thermal detection and pain thresholds were assessed at a constant room temperature of 22°C using a method of limits paradigm. For cold detection threshold (CDT) participants were instructed to click a mouse button when they perceived the temperature of the contact thermode decrease. Likewise for warmth detection threshold (WDT) participants responded to a perceived temperature increase. For cold/hot pain detection thresholds (CPT/HPT) participants were instructed to click the mouse button as soon as the temperature was perceived “painful”. The rate of change was 1°C/2 s and each variable was repeated three times. Thermal detection and pain thresholds were taken as the mean score obtained.

### Heat hyperalgesia curves

An 11-point Numerical Rating Scale (NRS) ranging from 0 (no pain) to 10 (worst pain imaginable) was used to assess the pain perceived by participants. To obtain a heat hyperalgesia curve^19^ subjects rated three different temperatures derived from the hot pain threshold. These consisted of three specific HPT, HPT + 2.5°C, and HPT-2.5°C delivered three times in a random order to each contact site. For each rating the chosen temperature was maintained for two seconds before returning to baseline (30°C) and the rate of change was 1°C/s.

### Pressure pain thresholds

Pressure pain threshold (PPT) was measured using a manual pressure algometer (Wagner Instruments, USA) with a contact diameter of 10 mm. The algometer was placed perpendicular to the surface at each of the three measurement sites and the pressure increased at a rate of approximately 0.5 Kg/Sec. The patient was instructed to indicate when the sensation of pressure first turned into pain at which point the algometer would be removed and the pressure reading taken. The average of three measurements were made at each site.

### Hyperalgesia

In order to sensitise the area of skin to facilitate the capsaicin-induced hyperalgesia [14], the contact thermode was applied to the CAPSITE and warmed to 41°C for five minutes. Following this capsaicin cream (Axsain 0.075% w/w, Bioglan LTD, New Zealand) was applied liberally to the area, ensuring a 1 cm overlap of the thermode footprint. A transparent film dressing (6 cm × 7cm Tegaderm™) was used to cover the capsaicin area and the subject rested for 30 min.

### Transcranial magnetic stimulation

TMS was delivered via a Nexstim NBS System (Nexstim, Helsinki) using a focal figure-8 coil with 9 cm external coil radius. Motor evoked potentials (MEPs) in response to TMS were recorded from the first dorsal interosseus muscle of the right hand using surface electromyography (EMG) electrodes (Ambu Neuroline 720). EMG signals were sampled at 3000 Hz and band-pass filtered between 10–500 Hz. In order to identify M1, motor thresholds and stimulation parameters, single pulse TMS was delivered at 60% stimulator output at various scalp locations over the inter-aural line of the left hemisphere of the brain. The site over which the largest responses were obtained (based on peak to peak MEP amplitudes) was marked on the scalp and taken as the site for M1 stimulation. Motor threshold was taken as the lowest stimulus intensity at which MEPs of amplitude of >0.05 mV were elicited in 5 out of 10 stimuli (mean threshold value = 37.8% (±3.6% s.e.m.). For rTMS, 100 stimuli were delivered over 10 s (10 Hz) each minute for 20 min (2000 stimuli) at 90% of the individual motor threshold level for each session with the coil positioned to direct stimuli anteriorly and perpendicular over the sulcus. For DLFPC stimulation, the TMS coil was positioned 5 cm anterior to M1 [19], and for occipital stimulation (OCC), over the occipital tuberosity at the back of the head with stimuli being directed anteriorly and parallel along the interhemispheric fissue [20].

### Psychological variables

Psychological factors including measures of emotion and catastrophising have been shown to be related to pain perception in clinical and experimental studies [21,22] and thus may account for some of the variability in pain reports. In the current study we used the Positive and Negative Affect Schedule (PANAS) [23] to assess emotion. The PANAS was designed to independently measure both positive affect (PA) and negative affect (NA) states. It comprises 20 adjectives (10 for each scale, e.g. Anger, Excited, Attentive) which participants are instructed to rate on a five-point scale from 1 ‘very slightly or not at all’ to 5 ‘very much’ (1: very slight or not reflecting the extent to which they currently felt the emotion. Total scores for both PA and NA may range from 10–50. Participants completed the PANAS at the start and end of each session. Pain Catastrophising, generally conceptualized as an exaggerated negative response to pain and its consequences, includes the cognitive processes of magnification, helplessness and rumination. We used the Pain Catastrophising Scale (PCS) [21] which asks participants to rate the degree to which they experience the 13 statements (e.g. I worry all the time whether the pain will end) when in pain on a 5-point scale from 0 ‘not at al’) to 4 ‘all the time’. Participants completed the PCS at the start of each session.

### Experimental design and protocol

A within-groups design was employed. There were two independent variables which included the brain site for rTMS, with four levels, no TMS, M1, DLPFC and OCC. The second independent variable was time, with three levels, PRE-CAP, POST-CAP (pre-TMS) and POST-TMS. Dependent variables included the hot and cold sensory and pain threshold temperatures, NRS scores obtained for the heat hyperalgesia temperatures, pressure scores; positive affect, negative affect and pain catastrophising scores.

Each participant took part in four conditions, with each visit at approximately the same time of day and a seven day interval between each condition. Separate investigators carried out the sensory measures (M.P.) and TMS (P.S.) in different laboratories The order of each rTMS condition was randomised and coded (no TMS was always the first) and only the investigator delivering rTMS knew which stimulus location was used for each session. Participants were instructed that on session two, three and four they would have TMS at three different brain sites. No more information was given about site locations or the impact each site location may have on subjective pain.

Each session followed the same protocol (with the exception of the first, no TMS session). Subjects were seated comfortably in an air-conditioned calm environment. The contact thermode was attached to CAPSITE and the area was outlined with a non permanent marker pen and QST and pressure pain measures were carried out (PRE-CAP time point). This procedure was then repeated on the clCAPSITE and then the nrCAPSITE. The contact thermode was then reapplied to the CAPSITE and the protocol for inducing heat hyperalgesia was carried out as described previously. After careful removal of capsaicin the contact thermode was reapplied to the CAPSITE and QST and pressure pain measures were repeated (POST-CAP time point). On completion capsaicin cream was reapplied and covered for precisely 30 minutes. For session one (no TMS) participants remained in the sensory motor laboratory. On sessions two, three and four participants went to the TMS laboratory for rTMS. Following rTMS participants returned and, after removal of the capsaicin, sensory testing was repeated at the CAPSITE, followed by the two non-capsaicin sites (POST-TMS time point). Each session lasted approximately 3 hours and each stage in the protocol was carefully timed for each subject to minimize variation between sessions.

### Data analysis

All results are expressed as mean (±s.e.m. except where stated otherwise). Data were analysed using Excel (Microsoft, USA) and SPSS (v20, IBM, USA). After confirming that the data variables were normally distributed (Kolmogorov-Smirnov test) within-within subjects repeated measures ANOVA were used to assess the effect of rTMS and time on the dependent variables at each time point for each contact site. Where a significant rTMS/time interaction was found post-hoc Bonferroni corrected paired t-tests were conducted to further characterise any significant differences between each of the rTMS sites. Repeated measures ANOVA was used to assess PANAS-PA at each time point for each contact site. PANAS-NA was not normally distributed, thus Kruskall-Wallis test was used to evaluate differences across conditions at each time point. One way ANOVA tested for differences in PCS score at baseline and for each contact site.

## Results

**Figure 1:**
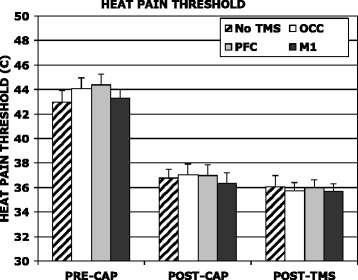
**Heat Pain Thresholds (HPT) for skin sites pre- and post-capsaicin and post-TMS.** There was a highly significant decrease following capsaicin application for all prospective TMS sites. rTMS had no significant effect on HPT during the second capsaicin application (OCC = occipital site; PFC = dorsolateral prefrontal cortex site; M1 = primary motor cortex site).

**Figure 2:**
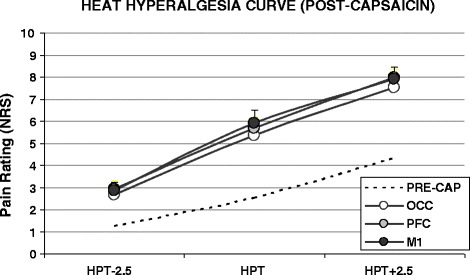
**Heat hyperalgesia curves for the post-capsaicin phase.** Capsaicin induced an approximate doubling of the perceived pain reported at temperatures around the HPT (dashed line = average pre-capsaicin curve). There were no differences between the prospective rTMS conditions.

Topical capsaicin application was well tolerated by subjects, although there was a progressive increase in subjective pain over the course of 30 min (mean NRS at 5, 15 and 30 min were 0.3 (±0.2), 3.9 (±1.2) and 6.6 (±1.8) respectively). QST values for the CAPSITE are shown in Table 1. There was no significant effect of capsaicin application on thermal sensory thresholds. In contrast HPT temperature was dramatically reduced following capsaicin (Figure 1) by 6.4-7.2°C (range of means; p < 0.0001) and this effect was reproducible over repeated sessions at the same site one week apart (Figure 1), i.e. for each prospective rTMS condition. There was also a trend for a reduction in CPT temperature at the CAPSITE (Table 1, average mean changes 0.9-3.4°C, differences n.s.). Capsaicin application had no effect on QST responses at either the nrCAPSITE or clCAPSITE (see Additional file 1: Tables S1 and S2). Following the second capsaicin application (with rTMS), there was a tendency for the HPT to be further decreased, but no evidence for any effect of rTMS (Figure 1). Similarly, rTMS had no effect on QST responses at either of the non-capsaicin sites (see Additional file 1: Tables S1 and S2). The heat hyperalgesia curves for the CAPSITE (Figure 2) demonstrated an approximate doubling in the NRS scores for the perceived pain at the HPT following capsaicin application (from 2.67 (±0.35) to 5.63 ± 0.57), with similar responses for each repeated measure (pre rTMS condition). This is despite the fact that the HPT temperatures were substantially lower following capasaicin application, demonstrating a strong and reproducible hyperalgesic effect.

**Table 1:** **QST and heat hyperalgesia (HYP) responses**

	**PRE-CAP**				**POST-CAP**				**POST-TMS**			
	***NO TMS***	***OCC***	***M1***	***DLFPC***	***NO TMS***	***OCC***	***M1***	***DLFPC***	***NO TMS***	***OCC***	***M1***	***DLFPC***
***CDT (°C)***	30.2 (.25)	30.4 (.32)	29.5 (.57)	30.4 (.32)	28.4 (.58)	27.7 (.82)	27.5 (.87)	27.1 (.78)	26.3 (.93)	28.0 (.61)	25.3 (1.14)	26.0 (.96)
***WDT (°C)***	33.5 (.71)	34.1 (.34)	34.2 (.30)	33.5 (.12)	34.4 (.20)	35.0 (.46)	34.5 (.59)	35.0 (.21)	34.4 (.15)	34.5 (.49)	34.3 (.25)	34.3 (.20)
***CPT (°C)***	17.4 (2.10)	13.0 (1.83)	13.0 (1.70)	15.6 (1.97)	8.3 (1.87)	12.1 (2.20)	9.8 (1.84)	12.5 (1.75)	5.9 (1.85)	8.1 (2.35)	8.3 (1.75)	6.4 (1.54)
***HPT (°C)***	42.9 (.96)	44.1 (.88)	44.4 (.88)	43.3 (.75)	36.8 (.71)	37.0 (.89)	37.0 (.86)	36.4 (.82)	36.1 (.89)	35.7 (.67)	36.0 (.65)	35.7 (.62)
***HYP- (NRS)***	2.2 (.21)	1.1 (.12)	1.5 (.26)	1.2 (.18)	4.5 (.51)	2.7 (.50)	2.9 (.41)	2.8 (.40)	4.0 (.48)	3.5 (.45)	2.9 (.38)	2.1 (.34)
***HYP0 (NRS)***	3.9 (.39)	2.7 (.36)	2.8 (.37)	2.5 (.34)	6.9 (.64)	5.3 (.58)	5.7 (.50)	5.9 (.59)	7.8 (.36)	6.7 (.46)	6.1 (.54)	5.1 (.63)
***HYP + (NRS)***	6.0 (.50)	5.4 (.58)	5.2 (.44)	4.3 (.46)	8.6 (.57)	7.5 (.51)	8.0 (.41)	7.9 (.58)	9.4 (.20)	8.7 (.29)	8.8 (.34)	7.9 (.57)

QST and heat hyperalgesia (HYP) responses for the capsaicin site at baseline, post-capsaicin and post combined capsaicin/rTMS treatment (CST = cold sensory threshold; HST = heat sensory threshold; CPT = cold pain threshold; HPT = heat pain threshold; HYP- = HPT – 2.5°C; HYP0 = HPT; HYP + = HPT +2.5°C).

**Figure 3:**
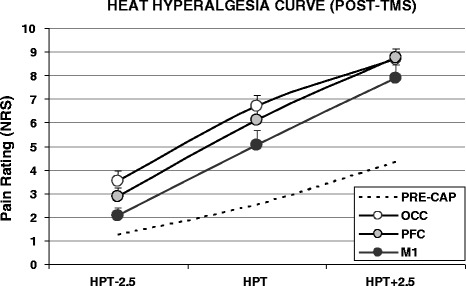
**Heat hyperalgesia curves obtained following combined capsaicin/rTMS treatment.** Subjects reported significantly reduced pain scores following for the M1 condition compared to both non-motor rTMS sites (dashed line = average pre-capsaicin curve).

Following repeated capsaicin application (during which rTMS was delivered) the heat hyperalgesia curves for the three rTMS conditions showed a divergence (Figure 3). There was a significant interaction between rTMS condition and time for the perceived pain ratings at HPT-2.5°C (F_(4,52)_ = 3.11, p < 0.001) and HPT (F_(4,52)_ = 4.45, p < 0.001) but not at HPT + 2.5°C (F_(4,52)_ = 2.10, p = 0.139). NRS pain reports were significantly lower for the M1 condition (despite the fact that HPT temperatures were similar to the two other rTMS conditions (Figure 1)). Subjects reported consistently lower NRS scores following rTMS of the DLFPC compared to OCC stimulation, but the differences were not significant. No changes in the hyperalgesia curves were observed at the non-capsaicin sites following rTMS.

**Figure 4:**
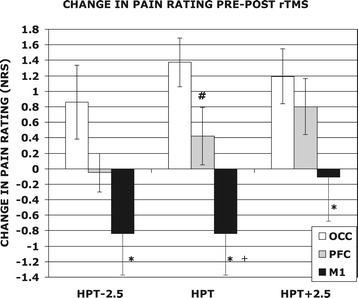
**Mean individual change in NRS of the heat hyperalgesia test for post-capsaicin vs post-TMS conditions.** M1 scores were significantly lower than OCC for all temperatures (*p < 0.03), and lower than PFC at the HPT (+p = 0.023). The PFC condition was consistently lower than OCC and this was significant at the HPT (#p = 0.033).

Figure 4 shows the mean difference in NRS scores of the POST-CAP vs POST-TMS heat hyperlagesia curves for each rTMS condition. Following capsaicin application with OCC stimulation, pain scores were significantly higher than for the first capsaicin application (mean increase = 1.37 (±0.31) at HPT (p < 0.001)). In contrast, when subjects received DLFPC stimulation there was lesser or no change in pain scores for the equivalent temperature (+0.42 (±0.38) at HPT, difference n.s.), with smaller effects seen at the lower temperature levels. Most strikingly, combined capsaicin and rTMS of M1 resulted in a *decrease* in the pain provoked by temperatures around the HPT (−0.84 (±0.53) at HPT, difference n.s.). This equates to a −24.7% pain reduction compared to OCC (p < 0.001) and a −18.3% reduction compared to the DLPFC condition, and again this effect was more apparent at lower temperatures (Figure 4).

There were no significant differences in positive affect (PA) (F_(3,52)_ = 1.94, *ns*) between the ‘no rTMS’ and three rTMS conditions, or between the start and end of each session (F_(1,52)_ = 3.11, *ns*) (see Additional file 1: Table S3). Similarly, non-parametric tests did not reveal any significant differences in negative effect (NA) between ‘no rTMS’ and three rTMS conditions, or from the start to end of each session. In addition, scores on the PCS were not significantly different between the no rTMS and three rTMS conditions (see Additional file 1: Table S4). Participant scores on these measures were consistent with available normative data in healthy populations [21,23] for PANAS and PCS respectively. However, given the relative lack of variability in scores across conditions and time in the current study, these data were not included in any further analysis.

## Discussion

This study found that the hyperalgesic effects of capsaicin application can be ameliorated by rTMS application over the cortex, and this is most effective following stimulation over the primary motor regions associated with the affected area. Capsaicin is believed to cause hyperalgesia via activation of nociceptive afferent C-fibres [24] and this effect is enhanced when the skin is subjected to a period of heat sensitization [14]. Unlike previous studies which have used topical capasacin alone to induce hyperalgesia, our subjects reported significant pain during application (although this resolved very quickly following its removal (i.e. during QST testing)). Despite this, capsaicin application had no effect on warmth or cold detection, or pain pressure thresholds, but profoundly reduced heat pain threshold temperature (by some 7°C) and to a lesser extent cold pain threshold (approx 2°C). This effect was consistent and reproducible over several weekly sessions, as demonstrated previously [15].

We compared responses to high frequency rTMS over M1 and DLFPC sites to a “control” site over the occipital fissure. We chose this site as most forms of sham TMS are easily unblinded due to reduction in scalp sensations associated with the sham coil. Occipital rTMS has been used as a control in a number of previous studies [20,25] and was well tolerated, with our participants reporting similar sensations as for treatment over M1, with the only difference being the occasional transient visual change (usually expressed as a slight increase in perceived light intensity). Therefore we believe that the OCC condition was an appropriate control comparison for the active rTMS sites.

We found that rTMS had no effect on any of the detection or pain threshold values associated with capsaicin application, either at the site of capsaicin application or on control sites. This somewhat contradicts previous findings. Thus Nahmias et al. [13] and Grueff-Guerro et al. [12] both found that rTMS over right hemisphere DLPFC and M1 resulted in a reduction in cold pain threshold temperature, whereas Borckhardt et al. [26] found similar effects with left hemisphere DLFPC stimulation. With regard to capsaicin-induced pain studies, rTMS of contralateral M1 [16] and left DLPFC [27] were found to reduce the pain associated with topical capsaicin application based on VAS scores. In this respect, our findings are in accordance since we found that the perceived pain associated with the HPT was significantly lower following rTMS over M1 compared with the OCC site. The subjective responses to heat pain were further refined by using a heat hyperalgesia curve, with responses obtained above and below HPT [28]. This confirmed the clear analgesic effect of M1 stimulation, especially at temperatures below HPT, and further demonstrated clear differences between M1 and DLFPC. The convergence of pain responses between rTMS conditions for the HPT +2.5C measure presumably reflects a ceiling effect of the analgesic response.

A number of possible mechanisms for the analgesic effects of primary motor cortex rTMS have been proposed involving activation of pain-modulation systems via direct or indirect means [29]. These could include activation of corti-cortical pathways projecting to brain areas associated with pain processing such as thalamus, anterior cingulate, insula, primary and secondary somatosensory cortices, periaquaductal grey and rostral ventromedullary areas [4,30‐32]. Indeed, these areas have been shown to be activated during heat induced pain from capsaicin sensitive skin [33‐36]. An indirect regulation of the same region via the thalamus appears equally possible. In particular TMS of M1 has been shown to result in changes in activity of the ventrolateral thalamus [30] and this area is believed to have a role in sensory function [37]. In this context it is interesting to note that Ohara et al. [38], using stereotactic microstimulation of various thalamic regions in conscious subjects, found that similar areas represented thermal and pain sensations.

In central post stroke pain, the effect of TMS of M1 on pain is critically dependent on the integrity of cortico-thalamic and thalamo-cortical connections [39]. Recently, indirect evidence has emerged that an additional mechanism, modulation of the descending pathways, can also play a role, as demonstrated for epidural motor cortex stimulation induced analgesia [40]. Onesti et al. [41] showed that during TMS of M1 for pain relief in diabetic neuropathy the nociceptive R III reflex was reduced, and this is thought to represent an inhibitory action from descending neuromodulatory pathways. We found that M1 stimulation had a significantly greater analgesic effect than that of DLFPC, however the close association between prefrontal and motor areas is well recognized. With regard to capsaicin-induced pain, Fierro et al. [27] found that both MEP amplitude and intracortical inhibition of M1 were reduced, and that left DLPFC rTMS resulted in both an analgesic effect and a normalisation of cortical excitability measures, suggesting a causal association between these phenomena. Moreover, Tamura et al. [16], using positron emission tomography scanning, found that the benefits of M1 rTMS from capsaicin-induced pain were correlated with a decrease in regional cerebral blood flow to medial prefrontal areas and an increase in blood flow to anterior cingulate. Regarding the lack of a strong analgesic effect associated with DLFPC stimulation, it must be acknowledged that a limitation of this study is that we did not use MRI-navigated TMS, and so can be less certain of the targeting of our rTMS [19]. However, we believe that this is unlikely to entirely explain the reduced effectiveness compared to M1 stimulation, since other studies have used the same reference site for DLFPC targeting [20]. Moreover, we did find a small but significant analgesic effect of pre-frontal when compared to OCC stimulation on pain responses at the HPT.

## Conclusion

In healthy subjects exposed to topical capsaicin-induced hyperalgesia, rTMS over the primary motor cortex resulted in a significant analgesic effect compared to other cortical areas. There are clear limitations in extrapolating the findings of acute pain models to the use of rTMS in the treatment of chronic pain syndromes since the neural changes associated with the two types of pain condition are likely to be different [42]. However our findings of reduced heat hyperalgesia following capsaicin application and rTMS of the primary motor cortex are interesting in the context of Lefaucheur et al. [43] findings that a similar pattern of stimulation in patients with chronic neuropathic pain resulted in improvements in both pain and warm sensory discrimination.

## Additional file

Additional file 1: Table S1. QST and heat hyperalgesia (HYP) responses for the near capsaicin (nrCAP) site at baseline and post combined capsaicin/rTMS treatment (CST=cold sensory threshold; HST=heat sensory threshold; CPT=cold pain threshold; HPT=heat pain threshold; HYP- = HPT – 2.5°C; HYP0 =HPT; HYP+ = HPT +2.5°C) (Mean/SEM). **Table S2.** QST and heat hyperalgesia (HYP) responses for the contralateral capsaicin (clCAP) site at baseline and post combined capsaicin/rTMS treatment (CST=cold sensory threshold; HST=heat sensory threshold; CPT=cold pain threshold; HPT=heat pain threshold; HYP- = HPT – 2.5°C; HYP0 =HPT; HYP+ = HPT +2.5°C) (Mean/SEM). **Table S3.** Pre and post positive and negative affect scores in the control and TMS conditions (Mean/SEM). **Table S4.** Pain Catastrophising Scale (PCS) scores in the control and TMS conditions (Mean/SEM). *df (3,55), *p>.*05.
