# Supplementary material for: Repetitive transcranial magnetic stimulation over primary motor vs non-motor cortical targets; effects on experimental hyperalgesia in healthy subjects
Source: BMC Neurol. 2014 Sep 4;14:166. doi: 10.1186/s12883-014-0166-3 (PMC4163168; doi:10.1186/s12883-014-0166-3)
Supplement: Additional file 1: Table S1. — QST and heat hyperalgesia (HYP) responses for the near capsaicin (nrCAP) site at baseline and post combined capsaicin/rTMS treatment (CST=cold sensory threshold; HST=heat sensory threshold; CPT=cold pain threshold; HPT=heat pain threshold; HYP- = HPT – 2.5°C; HYP0 =HPT; HYP+ = HPT +2.5°C) (Mean/SEM). Table S2. QST and heat hyperalgesia (HYP) responses for the contralateral capsaicin (clCAP) site at baseline and post combined capsaicin/rTMS treatment (CST=cold sensory threshold; HST=heat sensory threshold; CPT=cold pain threshold; HPT=heat pain threshold; HYP- = HPT – 2.5°C; HYP0 =HPT; HYP+ = HPT +2.5°C) (Mean/SEM). Table S3. Pre and post positive and negative affect scores in the control and TMS conditions (Mean/SEM). Table S4. Pain Catastrophising Scale (PCS) scores in the control and TMS conditions (Mean/SEM). *df (3,55), p>.05. [file 12883_2014_166_MOESM1_ESM.pdf]

	**PRE-CAP**				**POST-TMS**			
	NO TMS	*OCC*	*M1*	*DLFPC*	*NO TMS*	*OCC*	*M1*	*DLFPC*
CDT (°C)	30.3 (0.3)	29.9 (0.5)	29.7 (0.4)	29.9 (0.4)	30.1 (0.4)	29.7 (0.4)	29.5 (0.4)	29.2 (0.5)
WDT (°C)	34.3 (0.3)	34.7 (0.4)	34.1 (0.3)	34.8 (0.5)	34.5 (0.4)	34.8 (0.4)	34.3 (0.4)	34.9 (0.5)
CPT (°C)	19.4 (2.1)	17.0 (2.0)	15.3 (2.2)	15.3 (2.2)	15.9 (2.8)	17.5 (2.1)	14.8 (1.9)	15.3 (2.3)
HPT (°C)	42.6 (0.8)	44.1 (0.9)	43.6 (1.0)	43.6 (0.9)	42.8 (1.0)	43.4 (0.9)	43.6 (0.9)	43.2 (1.0)
HYP- (NRS)	2.1 (0.3)	1.6 (0.3)	1.8 (0.3)	1.6 (0.3)	1.8 (0.2)	1.8 (0.2)	1.5 (0.2)	1.5 (0.2)
HYP0 (NRS)	3.6 (0.4)	3.1 (0.5)	3.1 (0.5)	2.7 (0.3)	3.8 (0.5)	2.9 (0.4)	2.7 (0.3)	2.4 (0.4)
HYP+ (NRS)	5.9 (0.6)	5.3 (0.6)	5.2 (0.6)	5.2 (0.4)	6.1 (0.6)	5.1 (0.5)	5.2 (0.6)	4.5 (0.6)

Supp Table 1 QST and heat hyperalgesia (HYP) responses for the near capsaicin (nrCAP) site at baseline and post combined capsaicin/rTMS treatment (CST=cold sensory threshold; HST=heat sensory threshold; CPT=cold pain threshold; HPT=heat pain threshold; HYP**-** = HPT – 2.5°C; HYP0 =HPT; HYP+ = HPT +2.5°C) (Mean/SEM)

	**PRE-CAP**				**POST-TMS**			
	NO TMS	*OCC*	*M1*	*DLFPC*	*NO TMS*	*OCC*	*M1*	*DLFPC*
CDT (°C)	30.3 (0.2)	29.7 (0.4)	30.1 (0.3)	30.1 (0.3)	30.2 (0.3)	30.3 (0.3)	30.1 (0.4)	29.3 (0.6)
WDT (°C)	34.1 (0.2)	35 (0.6)	34.7 (0.4)	34.2 (0.4)	33.9 (0.2)	34.7 (0.4)	34.3 (0.5)	34.7 (0.5)
CPT (°C)	18.8 (1.8)	18.6 (1.8)	17.2 (1.9)	19.0 (1.8)	17.9 (2.0)	15.3 (1.8)	14.6 (2.1)	16.9 (2.0)
HPT (°C)	41.9 (1.0)	43.6 (0.9)	43.7 (0.8)	43.4 (0.7)	42.3 (0.8)	43.7 (0.8)	44.3 (0.7)	44.5 (0.7)
HYP- (NRS)	2.1 (0.3)	1.6 (0.2)	1.3 (0.2)	1.5 (0.3)	1.9 (0.3)	1.5 (0.2)	1.5 (0.3)	1.2 (0.2)
HYP0 (NRS)	3.9 (0.4)	2.9 (0.3)	3.1 (0.4)	2.8 (0.4)	3.2 (0.4)	2.3 (0.3)	3.0 (0.5)	2.3 (0.4)
HYP+ (NRS)	6.3 (0.4)	5.5 (0.5)	5.4 (0.5)	4.9 (0.5)	5.5 (0.4)	4.6 (0.4)	5.8 (0.7)	5.2 (0.5)

Supp Table 2 QST and heat hyperalgesia (HYP) responses for the contralateral capsaicin (clCAP) site at baseline and post combined capsaicin/rTMS treatment (CST=cold sensory threshold; HST=heat sensory threshold; CPT=cold pain threshold; HPT=heat pain threshold; HYP- = HPT – 2.5°C; HYP0 =HPT; HYP+ = HPT +2.5°C) (Mean/SEM)

	**PRE-TMS**				**POST-TMS**			
	NO TMS	*OCC*	*M1*	*DLFPC*	*NO TMS*	*OCC*	*M1*	*DLFPC*
POSITIVE AFFECT Mean(SEM)	28.36 (2.44)	26.64 (3.08)	24.85 (2.69)	24.14 (3.21)	26.78 (3.57)	25.93 (3.17)	28.00 (2.93)	24.93 (3.44)
NEGATIVE AFFECT Median(IQR)	12.50 (3.50)	11.00 (2.25)	10.00 (1.25)	10.00 (6.00)	12.00 (3.50)	11.00 (3.25)	10.00 (6.00)	10.00 (5.50)

Suppl Table 3 Pre and post positive and negative affect scores in the control and TMS conditions (Mean/SEM)

	NO TMS	*OCC*	*M1*	*DLFPC*	*F**
Rumination	7.93 (1.14)	6.14 (1.38)	5.64 (1.14)	6.14 (1.23)	.673
Magnification	5.07 (1.23)	3.36 (.78)	3.43 (.74)	3.21 (.73)	.719
Helplessness	8.86 (1.95)	6.5 (1.61)	5.93 (1.25)	6.07 (1.55)	.945
Total PCS	21.85 (4.16)	16.00 (3.63)	14.71 (2.85)	15.43 (3.38)	.860

Supplementary Table 4 Pain Catastrophising Scale (PCS) scores in the control and TMS conditions (Mean/SEM)

* df $_{(3,55)}$, p>.05
